# Some Inlay Points

**Published:** 1904-07

**Authors:** Alexander Jameson

**Affiliations:** Indianapolis, Ind.


### SOME INLAY POINTS.

By Alexander Jameson, D. D. S., Indianapolis, Ind.

(Written for *The American Journal of Dental Science.*)

*A Way to Provide Retention.*

If you have an inlay where you feel that the bottom should be rough for retentive purposes proceed as follows: Mix your body or a small part of it rather thin and let it extend almost to the edges of the cavity form. I do not know a better way to describe the proper thickness than to say to put on rather a thick coat of thinly mixed body. Dry so rapidly in furnace or on a hot slab of fire clay that you cause bubbles throughout the mass.

Bake this porcelain more than you do any subsequent layer and bake the subsequent layers as you would any porcelain to prevent bubbles.

When your inlay is finished, a few touches on the bottom with a corundum or "gem" stone will show a large number of pits or bubbles in exactly the place you wish your cement to adhere.

*Some Color Propositions.*

I wish to speak of two things which we as porcelain workers do not think of enough.

One is imitation, the other the attempt to reproduce certain natural conditions.

The tooth is translucent, consequently all our high fusing bodies are made translucent in the vain hope that we are reproducing a quality much to be desired. We make an inlay and try it and it is beautiful; then we set it with cement and all things are changed, because in using cement or anything else we have been inconsistent with our idea of reproducing conditions.

Did you ever put in a large distal porcelain filling in one of the six anterior teeth involving both labial and lingual walls, getting the color exactly right, only to find after it was in position that Ames' oxyphosphate or copper would have made a more acceptable filling, for you could have saved time on it? Here again is where our translucency comes in and comes in so hard that it defeats us because the inside of the mouth is not a very light place and our filling is so translucent that it lets out the darkness, as it were. Now these are examples of attempts to reproduce nature and the usual result is about the same.

Taking up the other side of the proposition, let us see what we can do in the way of imitation.

Taking up the other side of the proposition, let us see what we can do in the way of imitation.

There is an old saying that "beauty is only skin deep," and that's my idea about an inlay. It can be yellow or green or black on its inside if it only looks good on the outside. Why are the low fusing bodies more natural in appearance than the high fusing ones in the positions I have mentioned above? Because they are more opaque. They do not reproduce conditions, they imitate them, and the imitation is better than the attempted reproduction. Now, granting that we have reached the limit in reproducing conditions, and granting that we have nothing further to hope for in that line, it looks reasonable to me that we will have to advance in the direction of imitation, and I will predict that we will have an opaque porcelain rather than a translucent one as the next step in the line of dental art; or it may be a set of opaque enamels of different colors, and when we do have these the shadow problem and the cement problem, as a variable factor in the color problem, will have been solved.

